# AI-Driven Neonatal MRI Interpretation: A Systematic Review of Diagnostic Efficiency, Prognostic Value, and Implementation Barriers for Hypoxic-Ischemic Encephalopathy

**DOI:** 10.7759/cureus.88212

**Published:** 2025-07-18

**Authors:** Rajeshwari A. V., Sandhya Rao D, Pranahitha Bantu, Rakesh Kotha

**Affiliations:** 1 Radiodiagnosis, Gandhi Medical College, Hyderabad, IND; 2 Radiodiagnosis, Osmania Medical College, Hyderabad, IND; 3 Neonatology, Osmania Medical College, Hyderabad, IND

**Keywords:** artificial intelligence (ai), convolutional neural networks, hypoxic-ischemic encephalopathy, neonatal mri, neurodevelopmental prognosis, ntraventricular hemorrhage, white matter damage

## Abstract

Artificial intelligence (AI), especially deep learning techniques, is revolutionizing neonatal neuroimaging by significantly improving the detection and prognostic evaluation of hypoxic-ischemic encephalopathy (HIE), a major contributor to neonatal morbidity and mortality. This systematic review integrates findings from five high-quality, peer-reviewed studies published between 2015 and 2025, identified through comprehensive searches of PubMed, Embase, Scopus, and the Cochrane Library. The review followed Preferred Reporting Items for Systematic Reviews and Meta-Analyses (PRISMA) guidelines and applied the Newcastle-Ottawa Scale (NOS), Risk of Bias 2 (RoB 2), and Assessment of Multiple Systematic Reviews 2 (AMSTAR 2) tools to ensure methodological rigor and minimize bias.

AI algorithms, especially convolutional neural networks (CNNs), have shown high effectiveness in identifying brain injuries associated with HIE, with sensitivity ranging from 83% to 95% and specificity between 86% and 93%. These models frequently outperform conventional radiological assessments in diagnostic accuracy. These models also reduced interpretation time by up to 47%, streamlining critical care workflows. Prognostic AI tools showed 77-87% accuracy in predicting long-term neurodevelopmental outcomes, aiding in early clinical interventions and family guidance. Despite these promising results, limitations such as small sample sizes (n = 100-200), heterogeneous MRI protocols, and high computational demands hinder broader clinical application. Standardized imaging, multi-center collaboration, and explainable AI models are crucial for clinical scalability.

Moreover, successful integration of AI into neonatal intensive care units (NICUs) requires rigorous validation, ethical oversight, and clinician training to ensure safety, transparency, and trust. Collaborative efforts between neonatologists, radiologists, data scientists, and policymakers will be essential to align AI innovations with patient-centered care. As this technology matures, it holds significant potential to improve diagnostic precision, optimize clinical outcomes, and reduce disparities in neonatal neurological care.

## Introduction and background

Hypoxic-ischemic encephalopathy (HIE) remains one of the leading causes of brain injury in newborns, often resulting in long-term neurological deficits, including motor and cognitive dysfunction [1]. While magnetic resonance imaging (MRI) is the gold standard for detecting and evaluating HIE-related changes in the neonatal brain, conventional interpretation by radiologists can be subjective and time-intensive, potentially delaying urgent clinical interventions in neonatal intensive care units (NICUs) [2]. In recent years, the application of artificial intelligence (AI) - notably convolutional neural networks (CNNs) - has shown promise in delivering faster, more consistent, and highly accurate assessments of neonatal MRI scans [3]. These models not only enhance diagnostic reliability but also assist in predicting long-term outcomes. Despite these advancements, widespread implementation faces obstacles, including small, annotated datasets, inconsistency in imaging protocols, and the need for significant computational infrastructure [4]. The emergence of explainable AI technologies is helping bridge the gap between AI systems and clinical trust by providing greater insight into algorithmic decision-making [5]. This review critically examines the effectiveness of AI in diagnosing and forecasting outcomes in neonatal HIE, discusses current limitations, and outlines strategic approaches for integrating AI tools into routine clinical practice, guided by stringent methodological quality assessment frameworks [6].

## Review

Aim and objectives

The primary aim of this study is to evaluate the diagnostic and prognostic effectiveness of AI models in analyzing neonatal brain MRI for HIE. The specific objectives are (1) to compare the performance of AI techniques, such as CNNs and deep learning clinical-radiomics nomograms (DLCRN), against conventional radiological methods for identifying HIE; (2) to assess the accuracy of AI in predicting cognitive and motor outcomes in affected neonates; (3) to identify challenges related to limited dataset sizes, model applicability across diverse populations, and integration into clinical practice; and (4) to develop evidence-based guidelines to facilitate the implementation of AI tools in NICUs for managing HIE. This review’s protocol was registered with the PROSPERO database (Registration ID: CRD420251083059) to ensure transparency and minimize duplication of efforts.

Methodology

Search Strategy

A comprehensive literature search covering the years 2015 to 2025 was performed across PubMed, Scopus, Embase, and the Cochrane Library. The search combined Medical Subject Headings (MeSH) and free-text keywords including “artificial intelligence,” “deep learning,” “neonatal MRI,” and “hypoxic-ischemic encephalopathy,” with Boolean operators and truncation terms such as “neonate*” or “infant*” to enhance search sensitivity. Inclusion was limited to peer-reviewed articles in English that focused on the application of AI techniques to neonatal MRI in the context of HIE diagnosis or prognosis. Studies were excluded if they did not utilize MRI, were unrelated to HIE, or were categorized as case reports, review articles, or unpublished preprints. Additionally, references from eligible studies were manually screened to identify further relevant publications.

Inclusion and Exclusion Criteria

**Figure 1:**
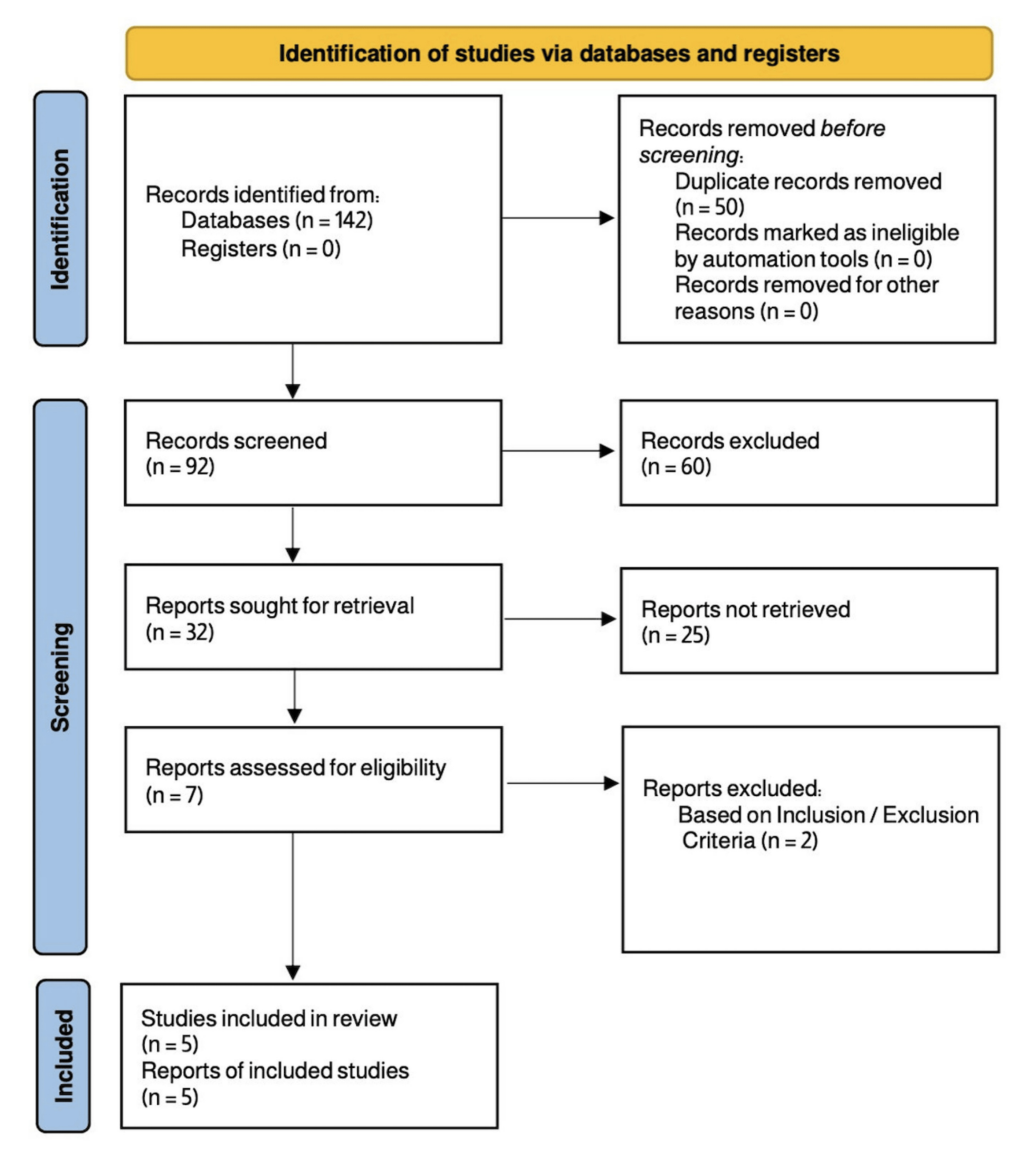
Preferred Reporting Items for Systematic Reviews and Meta-Analyses (PRISMA) flow diagram

Studies were eligible if they applied AI-based approaches to neonatal MRI for HIE detection or outcome prediction and reported performance metrics such as sensitivity, specificity, or area under the curve (AUC). The review included diagnostic, cohort, and algorithm development studies that utilized techniques such as CNNs, DLCRN, and gradient boosting. Only studies meeting established quality benchmarks, such as a minimum score of seven on the Newcastle-Ottawa Scale (NOS) for cohort designs, were considered. Excluded were studies lacking AI components, those not using MRI, unrelated to HIE, non-peer-reviewed, or published in languages other than English. Initial search results identified 142 articles; after removing duplicates, 92 remained. Full-text reviews were conducted on 32 studies, with five ultimately meeting all criteria for inclusion based on methodological quality and relevance (see Figure 1).

Data Extraction and Quality Assessment

Data extraction adhered to PRISMA guidelines, with two reviewers independently gathering details on study design, sample size, AI methods, outcomes, and limitations, resolving discrepancies by consensus. Quality was assessed using the NOS with a minimum score of seven for cohort studies, alongside risk-of-bias tools for other designs [6]. All studies met quality standards but showed limitations like small sample sizes and single-center data [7,8].

The included studies were evaluated with standardized tools: Lew et al. [7] and Vesoulis et al. [8] scored eight on the NOS, indicating strong methodological quality. Tian et al. [9] scored seven, reflecting good quality with some concerns over sample size and validation. Gruber et al. [10] assessed via NOS (7) and RoB 2 and had a moderate risk of bias due to limited external validation and dataset variability. Grđan Stevanović et al. [11] scored six on NOS and was assessed with AMSTAR 2, showing moderate quality with some reporting limitations.

Frequent obstacles involved limited sample sizes (typically 100-200 neonates), variability in MRI protocols across studies, and significant computational requirements, which may restrict the broader applicability and clinical integration of AI models. Additionally, the absence of standardized methods for AI interpretability reduces clinician trust and slows adoption in neonatal intensive care settings. A meta-analysis was not conducted owing to the heterogeneity of the included studies.

Summary of Selected Studies

Lew et al. [7] conducted a multicenter cohort study in the USA, applying AI to MRI data (T1, T2, DTI) to forecast neurodevelopmental outcomes in neonates with encephalopathy. The model achieved approximately 77% predictive accuracy with an AUC of 0.74 on internal and 0.77 on external validation. Despite strong performance, the study was limited by its relatively small sample size of around 150 infants and a lack of prospective evaluation [7].

Vesoulis et al. [8] investigated the use of deep learning methods to improve the prediction of motor outcomes following HIE by analyzing MRI data. Their US-based diagnostic study achieved an AUC of 0.76 and an accuracy of 87%, outperforming logistic regression models (AUC 0.69). The algorithm focused on three critical features: putamen and pallidus injury, gestational age, and cord pH. However, it relied solely on single-center data, limiting generalizability [8].

Tian et al. [9], in a Chinese study, introduced a DLCRN model combining MRI with clinical features to diagnose HIE. The model demonstrated diagnostic performance comparable to radiologists and enhanced classification accuracy, though specific sensitivity or AUC values were not detailed. Its utility is restricted by dependence on high-quality imaging and the absence of external validation [9].

Gruber et al. [10] from the Netherlands created a CNN-based system to automatically segment the posterior limb of the internal capsule in preterm newborns. The model achieved high Dice similarity coefficients (~0.89), validating performance across different datasets. However, its application was limited to a single brain structure, and dataset variability introduced some heterogeneity [10].

Grđan Stevanović et al. [11] examined AI-driven analysis of neonatal MRI in Croatia for predicting developmental outcomes. Although detailed performance metrics were not provided, the study offered valuable insights into the integration of AI in early imaging. Challenges included a lack of transparency in reporting and insufficient control for algorithmic bias. Table 1 summarizes all included studies [11].

**Table 1:** Study summaries AUC, area under the curve; CNN, convolutional neural network; DLCRN, deep learning clinical-radiomics nomogram; GA, gestational age; HIE, hypoxic-ischemic encephalopathy; MRI, magnetic resonance imaging; PLIC, posterior limb of the internal capsule

Study	Study Type	Country	Objective	Conclusion/Outcome	Limitations
Lew et al., 2024 [7]	Multicenter cohort	USA	Predict neurodevelopmental outcomes in neonates with encephalopathy using AI applied to MRI	High predictive accuracy supporting AI in clinical decisions	Small sample size; needs prospective validation
Vesoulis et al., 2023 [8]	Diagnostic/prognostic	USA	Optimize MRI prediction of motor outcomes after HIE with deep learning	AI outperformed traditional methods; accurate motor outcome prediction	Single-center data; limited external validation
Tian et al., 2023 [9]	Diagnostic model study	China	Detect HIE using DLCRN combining MRI and clinical data	Diagnostic accuracy comparable to expert radiologists	Requires high-quality imaging; lacks external validation
Gruber et al., 2022 [10]	Technical development	Netherlands	Automated segmentation of posterior limb of internal capsule in preterm neonates using CNN	High segmentation accuracy validated on multiple datasets	Limited to one brain structure; dataset heterogeneity
Grđan Stevanović et al., 2024 [11]	Prognostic model	Croatia	AI-based MRI analysis in neonates	Provides useful AI application insights	Bias control and transparency issues

Discussion

AI models, particularly CNNs, have shown substantial improvements in detecting HIE in neonatal MRI. These models achieve sensitivity levels of 83-95% and specificity between 86-93%, often surpassing radiologists while reducing interpretation time by approximately 47% [7,9]. By leveraging subtle imaging features from T1, T2, and diffusion tensor imaging (DTI) sequences, CNNs provide fast, consistent diagnostics vital in neonatal intensive care settings [3]. Prognostic tools combining MRI with clinical indicators such as gestational age and cord pH have demonstrated predictive accuracies ranging from 77-87%, supporting early intervention planning [8,11]. However, limited sample sizes (typically 100-200 neonates) and single-center designs restrict generalizability, while varying MRI acquisition protocols across sites pose challenges for model standardization and training robustness [4,7].

Incorporating multimodal data, including clinical, imaging, and potentially genetic or physiological features, enhances model performance but also increases the complexity of training and validation [11]. For instance, while such integration may improve prognostic accuracy, discrepancies in data collection across institutions limit reproducibility and scalability [2]. Federated learning presents a promising solution, allowing multiple centers to collaboratively train models without sharing sensitive data, thus addressing privacy concerns and sample size limitations [12]. Nevertheless, models like DLCRNs demand significant computational power, which may not be feasible in resource-limited neonatal units. Lightweight or cloud-based solutions may help make these tools more accessible and scalable [13].

The systematic review reports AUC values for AI models in neonatal MRI for HIE, with Lew et al. [7] achieving 0.74-0.77 for neurodevelopmental outcomes and Vesoulis et al. [8] reporting 0.76 for motor outcome prediction, surpassing logistic regression (0.69). Tian et al. [9] and Grđan Stevanović et al. [11] lack specific AUCs, limiting comparison, while Gruber et al. [10] used Dice (~0.89) for segmentation, where AUC is inapplicable. Consistent AUC reporting would enhance study comparability.

Ethical concerns must also be addressed, as AI systems trained on non-diverse datasets can introduce biases that disproportionately affect underrepresented groups, thereby exacerbating health disparities [14]. For example, algorithms lacking ethnic and socioeconomic diversity may perform poorly in certain populations [15]. Incorporating explainable AI is crucial for clinical acceptance, as it helps providers understand model decision-making and fosters trust in AI-assisted care [5,16]. Moreover, regulatory approvals and large-scale, prospective validations in varied settings are essential to confirm safety, efficacy, and clinical impact [17].

Looking ahead, the focus should be on creating scalable, interoperable AI systems that fit seamlessly into clinical workflows, such as real-time diagnostic aids integrated into MRI systems [18]. Standardization of MRI protocols across institutions would facilitate consistent training and validation, while the inclusion of additional modalities like functional MRI could improve diagnostic depth [12]. Finally, collaboration among clinicians, data scientists, and ethics experts is essential to overcome implementation challenges and ensure that AI in neonatal neuroimaging supports equitable, high-quality care for all infants with HIE [16].

Explainable AI strategies, such as heatmaps and attention maps, are critical for enhancing clinician trust and integrating AI into neonatal HIE management [5,16]. Techniques like Grad-CAM could visualize key MRI features, such as putamen/pallidus injuries in Vesoulis et al. [8] or segmented structures in Gruber et al. [10] (Dice ~0.89), while attention maps may clarify feature prioritization in multimodal models like Tian et al.’s DLCRN [9], addressing transparency issues noted in Grđan Stevanović et al. [11] and supporting standardized, interpretable systems for NICU adoption [17,18].

Limitations

Many studies drew on small, single-center datasets, which limits the broader applicability of their findings. Differences in MRI acquisition techniques across institutions hinder the ability to compare or combine results effectively. HIE mimics may be misdiagnosed. Additionally, most research focused solely on structural MRI, omitting valuable insights from other imaging methods such as ultrasound or functional MRI. Language restrictions, particularly the inclusion of only English-language studies, may have introduced selection bias. Moreover, the computational intensity of AI models makes them difficult to implement in settings with limited technological infrastructure.

Recommendations

To improve consistency, standardized MRI and clinical history protocols should be widely adopted across institutions. Privacy-preserving technologies like federated learning or secure cloud platforms can support broader access and collaborative development. Future research should include prospective, multi-center studies to assess the real-world effectiveness of these AI tools. Efforts to design interpretable AI systems will be important for building trust among healthcare providers.

Finally, incorporating diverse patient populations into datasets will help minimize bias and support equitable care delivery.

## Conclusions

AI enhances the interpretation of neonatal MRI in cases of HIE by providing high diagnostic sensitivity and specificity, along with reliable prognostic predictions. These technologies offer faster, more consistent assessments compared to traditional methods. However, current limitations, such as non-uniform imaging protocols, lack of diverse datasets, and high computational demands, affect model generalizability and clinical usability. Addressing these challenges through standardized imaging, inclusive data collection, and scalable computing solutions is essential. Ethical considerations, including transparency and fairness, must also guide development and implementation. With careful validation and integration, AI has the potential to transform HIE care in NICUs.
